# Deficiency of Parkinson’s disease-related gene *Fbxo7* is associated with impaired mitochondrial metabolism by PARP activation

**DOI:** 10.1038/cdd.2017.175

**Published:** 2017-10-13

**Authors:** Marta Delgado-Camprubi, Noemi Esteras, Marc PM Soutar, Helene Plun-Favreau, Andrey Y Abramov

**Correction to:** *Cell Death and Differentiation* (2017) **24**, 120–131; doi:10.1038/cdd.2016.104; published online 30 September 2016

This article was originally published under a Creative Commons Attribution-NonCommercial-NoDerivs 4.0 license, but has now been made available under a Creative Commons Attribution 4.0 (CC BY) license.

The PDF and HTML versions of the paper have been modified accordingly.

